# Patch detachment after mitral valve repair with posterior leaflet augmentation: a case report

**DOI:** 10.1186/s13019-015-0322-x

**Published:** 2015-09-12

**Authors:** Toshihito Gomibuchi, Tamaki Takano, Yuko Wada, Takamitsu Terasaki, Tatsuichiro Seto, Daisuke Fukui

**Affiliations:** Department of Cardiovascular Surgery, Shinshu University School of Medicine, Asahi 3-1-1, Matsumoto, Nagano 390-8621 Japan

**Keywords:** Ischemic mitral regurgitation, Cardiopulmonary arrest, Mitral valve repair

## Abstract

Mitral valve (MV) repair is indicated for patients with severe MR. We report a case of acute MR caused by patch detachment after posterior leaflet augmentation in MV repair. A 65-year-old male underwent MV repair with posterior leaflet augmentation and coronary artery bypass graft 1 month prior to this study. An inverted T-shaped incision was made on the posterior mitral leaflet (PML), and a piece of autologous fresh pericardium was sewn in the PML defect. Seven days after hospital discharge, he started feeling chest pain and presented with pulseless electrical activity. Ultrasonic cardiography showed severe mitral regurgitation (MR), which was suggestive of acute MR. We performed emergency reoperation. The edge of the autologous pericardial patch was detached from the anterior papillary muscle, and MV replacement was performed. He was discharged from the hospital 55 days after the reoperation and returned to his normal daily life. We conclude that avoidance of tension focalization during MV repair may be important.

## Background

Ischemic mitral regurgitation (MR) results from left ventricular remodeling after myocardial infarction and significantly affects prognosis. Mitral valve (MV) repair or replacement is indicated for patients with severe MR. Ring annuloplasty is most commonly used for MV repair [1] although long-term outcomes are often unsatisfactory [2, 3]. The use of standard surgical techniques in MV repair are still under debate; however, it is proposed that chordae cutting, papillary muscle approximation and leaflet augmentation combined with annuloplasty greatly improve clinical outcomes in MV repair [4–6]. We report a case of acute MR caused by patch detachment after posterior leaflet augmentation in MV repair.

## Case presentation

**Fig. 1:**
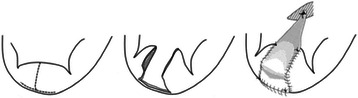
An inverted T-shaped incision was made in the posterior mitral leaflet (PML), and the edge of patch was directly fixed to the anterior papillary muscle

A 65-year-old male underwent MV repair with posterior leaflet augmentation and coronary artery bypass grafting (aorta-oblique marginal branch-posterior descending with saphenous vein graft) in another hospital 1 month prior to this study. Preoperative transthoracic echocardiography showed dilated left ventricle (LV) with inferoposterior wall akinesis and over all impaired LV function with 39 % of ejection fraction. There was severe mitral regurgitation (MR) with 19.6 mm of tethering height, and coaptation depth was 13 mm. Augmentation of the posterior mitral leaflet (PML) was chosen because there was fear of MR recurrence if only mitral annuloplasty was done. An inverted T-shaped incision was made on the PML, and a piece of autologous fresh pericardium was cut to a pentagonal shape and sewn in the PML defect. The edge of the patch was directly fixed to the anterior papillary muscle by pericardium pledgeted single suture with 4–0 polypropylene. Annuloplasty was performed with Physio II 32 mm (Edwards Lifesciences, Inc, Irvine, CA, USA) (Fig. 1). Intraoperative transesophageal echocardiography showed no MR, but postoperative transthoracic echocardiography showed mild-to moderate MR in the medial scallop. He was discharged from the hospital 21 days after the surgery.

Seven days after discharge, he started feeling chest pain while driving and called the emergency services. When they arrived, he had pulseless electrical activity. Cardiopulmonary resuscitation was started immediately and adrenaline and atropine were administered twice. He regained spontaneous circulation after two minutes of resuscitation and then transferred to our hospital by a helicopter after tracheal intubation. Echocardiography showed prolapsing pericardium attached to the PML and massive MR. Coronary angiography revealed saphenous vein graft occlusion. Percutaneous coronary intervention was performed on the occluded graft; however, his blood pressure gradually decreased and he developed anuria, even with inotropic support using dopamine, dobutamine and intra-aortic balloon pump (IABP). Repeated ultrasonic cardiography showed severe hypokinesis in the inferoposterior wall of the left ventricle, equivalent to that before coronary intervention, and pulse Doppler showed severe MR with rapid decrease of regurgitation flow “cut off sign” in the late systolic phase, which was suggestive of acute MR.

**Fig. 2:**
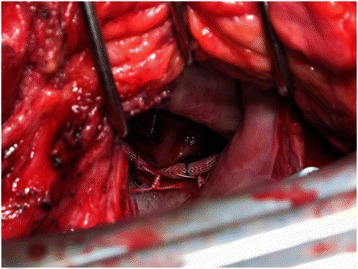
Operative view of the annuloplasty ring and the autologous pericardial patch. The patch detached from the papillary muscle and prolapsed

Emergency surgery was performed. Cardiopulmonary bypass (CPB) was initiated with cannulation of the ascending aorta, superior vena cava and right femoral vein. The ascending aorta was cross-clamped and a left-sided atriotomy was made. The mitral annuloplasty ring remained fixed at the annulus; however, the edge of the autologous pericardial patch was detached from the anterior papillary muscle so that the posterior leaflet extensively prolapsed toward the left atrium (Fig. 2). Papillary muscle rupture was not observed and there was no vegetation or abscess on the mitral annuloplasty ring, mitral annulus and autologous pericardium. However, we found necrosis in the residual PML, and it seemed impossible to perform either of re-repair or valve replacement with leaflet preservation. After removal of the prosthetic ring, the anterior and posterior leaflets were resected. Mosaic 25 mm (Medtronic, Minneapolis, MN, USA) was placed on the mitral annulus. CPB was weaned off with high dose catecholamine and IABP support. Continuous hemodiafiltration was started postoperatively to treat acute renal failure and maintenance hemodialysis was initiated. IABP support was required for 3 days, and the patient was extubated on postoperative day 4. He was discharged from the hospital after 55 days and returned to his normal daily life without neurological complications.

## Discussion

Although MR is one of the severe complications after myocardial infarction, the most preferable surgical procedure for ischemic MR has not been established [1, 2]. MV repair has a higher recurrence rate than MV replacement, although a recent meta-analysis showed lower perioperative mortality and better survival with MV repair at 5 years compared with that of MV replacement [1, 2]. There are various surgical procedures to improve surgical outcome in MV repair. Anterior leaflet augmentation was reported 81 % of actuarial freedom from moderate or greater MR in 25 patients [5]. An animal study revealed that posterior leaflet augmentation, attained more coaptation than annuloplasty alone [4]. Here we report a case and highlight the risk of sudden patch detachment after MV repair with posterior leaflet augmentation.

Generally, leaflet preservation during the MV replacement was recommended for ischemic mitral regurgitation [7]. Preservation of subvalvular apparatus and valve-ventricular interaction was proved to play an important role in preserving left ventricular regional wall motion and global function. Leaflet preservation may help prevent serious complications after MV replacement [8]. However, it was impossible to perform re-repair or valve replacement with leaflet preservation because there was necrosis in residual posterior leaflet in the presenting case.

Cardiac arrest caused by severe MR because of detachment of artificial structures from the papillary muscle is extremely rare, and we were unable to find any other report in which the patient was successfully treated with emergency surgery. The cause of cardiac arrest in our case could have been because of either graft occlusion of the previous coronary artery bypass or acute MR. However, the hemodynamic status was unstable after successful coronary intervention and ultrasonic cardiography showed acute MR. Therefore, acute MR was considered to be the primary cause of cardiac arrest. There is a possibility that cardiopulmonary resuscitation caused the patch detachment after cardiac arrest because of bypass occlusion. This is also extremely rare, but it is worth being aware of the risk of chest compression that causes patch detachment after mitral repair.

In this case, the center of the posterior leaflet was incised in a T-shape and an edge of the pericardial patch, which was sutured to the incised posterior leaflet, was directly fixed to the papillary muscle with a single point in the initial surgery. Therefore, there may have been excessive tension focalization on the patch edge during systole, causing the patch to detach suddenly from the papillary muscle leading to acute MR and cardiopulmonary arrest. Thus, it may be important to avoid tension focalization during MV repair.

## Conclusion

We report a case of patch detachment after MV repair with posterior leaflet augmentation, which highlights the potential importance of avoiding tension focalization during MV repair.

## Consent

Written informed consent was obtained from the patients for publication of this Case report. Copies of the written consent forms are available for review by the Editor-in-Chief of this journal.

## Abbreviations

MV: Mitral valve
PML: Posterior mitral leaflet
MR: Mitral regurgitation
IABP: Intra-aortic balloon pump
CPB: Cardiopulmonary bypass
